# Art or Science? An Evidence-Based Approach to Human Facial Beauty a Quantitative Analysis Towards an Informed Clinical Aesthetic Practice

**DOI:** 10.1007/s00266-017-1032-7

**Published:** 2018-01-08

**Authors:** Harpal Harrar, Simon Myers, Ali M. Ghanem

**Affiliations:** Academic Plastic Surgery Group, Blizard Institute, Barts and the London School of Medicine and Dentistry, Queen Mary University of London, 4 Newark Street, London, E1 2AT UK

**Keywords:** Beauty, Facial measurements, Anthropometry, Facial beauty, Aesthetic, Quantitative analysis

## Abstract

**Background:**

Patients often seek guidance from the aesthetic practitioners regarding treatments to enhance their ‘beauty’. Is there a science behind the art of assessment and if so is it measurable? Through the centuries, this question has challenged scholars, artists and surgeons.

**Aims and Objectives:**

This study aims to undertake a review of the evidence behind quantitative facial measurements in assessing beauty to help the practitioner in everyday aesthetic practice.

**Methods:**

A Medline, Embase search for beauty, facial features and quantitative analysis was undertaken.

**Selection Criteria:**

Inclusion criteria were studies on adults, and exclusions included studies undertaken for dental, cleft lip, oncology, burns or reconstructive surgeries. The abstracts and papers were appraised, and further studies excluded that were considered inappropriate. The data were extracted using a standardised table. The final dataset was appraised in accordance with the PRISMA checklist and Holland and Rees’ critique tools.

**Results:**

Of the 1253 studies screened, 1139 were excluded from abstracts and a further 70 excluded from full text articles. The remaining 44 were assessed qualitatively and quantitatively. It became evident that the datasets were not comparable. Nevertheless, common themes were obvious, and these were summarised.

**Conclusion:**

Despite measures of the beauty of individual components to the sum of all the parts, such as symmetry and the golden ratio, we are yet far from establishing what truly constitutes quantitative beauty. Perhaps beauty is truly in the ‘eyes of the beholder’ (and perhaps in the eyes of the subject too).

**Level of Evidence V:**

This journal requires that authors assign a level of evidence to each article. For a full description of these Evidence-Based Medicine ratings, please refer to the Table of Contents or the online Instructions to Authors www.springer.com/00266.

## Introduction

Facial aesthetic treatments have a significant influence on the individual and her perception of life. Differences in facial appearance provide individuality and are readily noticeable. The increasing appreciation of facial volume and tissue change has allowed the innovation and widespread use of fillers and the evolution of filling techniques, particularly fat grafting [1].

Demand for facial aesthetic treatments has increased in the last 20 years [2, 3]. With this increase, it is becoming more important for the clinician delivering these treatments to understand what constitutes beauty and what motivates the patient to strive for beauty. Our self-perception of beauty has an impact on our everyday lives [4]. Others perceive a beautiful person to be more intelligent, sociable, friendlier and more desirable [5].

Many scholars throughout the centuries have debated what comprises beauty and indeed how to measure it in a standardised reproducible way [6]. Despite this centuries-old debate, there does not appear to be a validated, widely used set of evidence-based rules or measurements that can influence clinical practice. Understanding quantitative and objective features that constitute facial beauty is complex and confounded by multiple elements including society, culture, age and ethnicity [7]. Some argue that beauty is a myth and not reality and that the perception is learned and not developmental [8], and yet others argue that the perception of beauty is an innate developmental or biological ability [9]. Over the past few decades, the advancement of computer technology and computational capability may play a role in facilitating the assessment or evaluation of beauty. Differences in perceptions of facial aesthetics between professionals and patients have been well documented [10]. Pre-planning, managing expectations and discussion of potential sequelae are already established protocols used by clinicians for a successful outcome for the patient. If standardised facial measurements could be incorporated into this process, it might allow the measurement of outcomes, have the potential to change the dynamics of a consultation and act as a useful consultation tool, to help manage expectations.

These measures based on evidence could be used as standards to guide the clinician. Based on the PICO framework [11], this study will aim to answer this research question—in the treatment of adults requesting facial aesthetic improvement, is there an evidence-based approach in quantitatively assessing beauty that is useful in everyday aesthetic practice?

## Methods

A literature review was undertaken using Pubmed Medline, Medline Ovid, and EMBASE. Date limits were applied from 1970 to April 2017, and publications in English, humans and in peer-reviewed journals were included, with exclusions for abstracts presented at conferences. The search strategy was devised using three main concepts: (1) beauty AND (2) facial features AND (3) quantitative analysis (including terms proportions, distance, dimensions, length, height and width). Both thesaurus terms and text words (words or phrases appearing in the title or abstract of references) were identified for each concept. A manual check was undertaken given the sensitive nature of the search strategy used (use of quantitative analysis to aid in plastic or cosmetic surgical procedures to correct facial deformities or conditions); the search strategy is available.

## Inclusions

Research papers, where adults were subjects, seeking facial aesthetic therapies or facial assessments were considered. Outcome variables of measured beauty parameters, facial measurements, ratios of measurements of the face, comparison of facial parameters were included.

Exclusions were applied for facial measurements undertaken for research on cadavers, burns and trauma victims seeking aesthetic treatments. Exclusions were also applied where plastic and reconstructive or dental surgery would have been the predominant procedure.

## Results

**Fig. 1:**
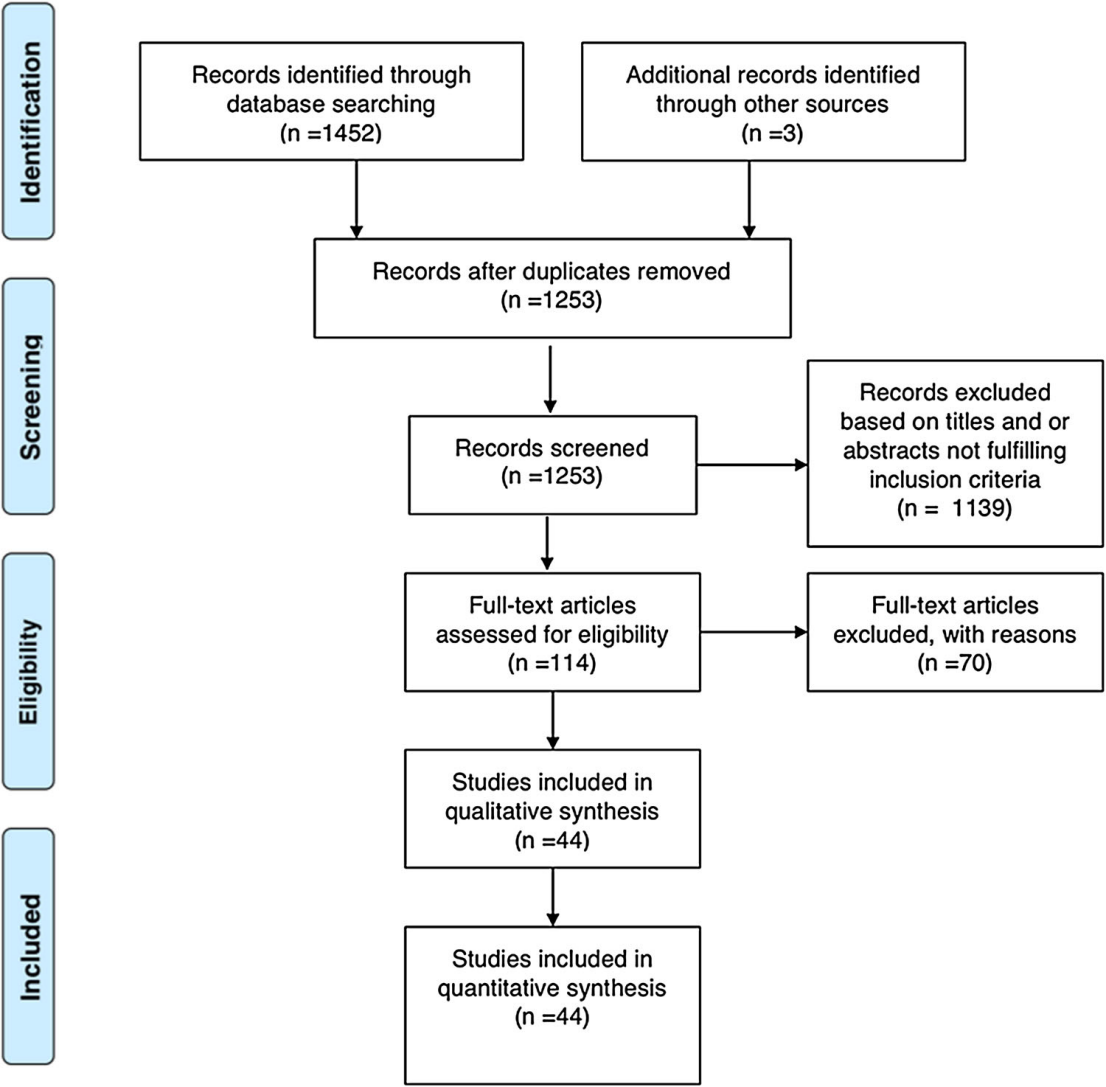
Study protocol flow diagram

A total of 182 entries had been considered as duplicates from the search of 1455. The search criteria did not fully exclude articles with patients who were children or adolescents, and these were further excluded after reviewing the abstracts. Table 1 lists excluded articles and reasons for their exclusion. Of the remaining 44 studies, the full articles were extracted and checked. These were further scrutinised for their methodology and outcomes data. Due to the diversity of the types of studies, combining them was not appropriate statistically although some grouping was possible according to common themes (Fig. 1, Table 2).

**Table 1:** Reasons for exclusion of full text articles

Reason for exclusions	Number
Dentofacial surgical correction/Le fort osteotomy orthognathic	14
Psychological effects of beauty/personality and beauty/brain effects on beauty	12
Inappropriate for other reasons	8
Orbital surgery/ear placement in reconstruction	6
Skeletal analysis	5
Cleft lip palate and surgery	3
Adolescent or child after manual records reviewed	3
Cancer surgery	3
Cosmetic or cosmeceuticals	3
Comparison of different fillers	2
Endoscopic lifting surgery	2
Burns victims/trauma victims	2
Qualitative measurements of facial aesthetic outcomes	2
DNA forensic analysis	2
Portrait painting theories	2
Cadaver	1
Total	70

**Table 2:** Summary of trials [12–41]

Article	Year of study	Sample size	Measures rating beauty	Type of study/level of evidence	Outcome	Comments
Liu et al. [12]	2017	360	Distances and angles	Computation based on photographs level III	Measurements do not have a normal distribution, no constant relationship of proportionality	An in-depth mathematical analysis of distances and angles
Heidekrueger [13]	2017	1011	Lip ratio preference	Survey level IV	Lip ratio of 1.0:1.0 was most attractive	Survey of surgeons’ preference
Koidou et al. [14]	2017	193	Angulation of smile	Case control level III	Smaller mean angulation of smile more aesthetically pleasing	
Jang et al. [61]	2017	93	Measurements from three-dimensional sampling	Case control level III	Longer face smaller lower lip and chin preferred. deviation from golden ratio	Korean population
Popenko et al. [15]	2017	20 digital images altered to create 100 faces	Lip surface area and lower/upper lip ratio	Survey level IV	53.5% increase in surface area and 2:1 ratio of lower to upper lip more attractive	Age 18–25 white female faces
Benslimane et al. [16]	2017	450 photos 1000 portraits 339 patient photos	Eye fissure frame ratio or ‘Frame concept’	Cross-sectional level IV	Frame height is inversely proportional to attractiveness and narrower eye fissure frame more attractive	Novel idea of ‘Frame concept’
Melo et al. [17]	2017	30	Harmony of features	Cross-sectional level IV	Subjective influence on assessment of attractiveness	Subjective facial analysis criteria used. photographs rated by 50 evaluators
Kaipainen et al. [18]	2016	59	Effect of regional facial asymmetry on attractiveness	Observational level IV	Attractiveness not influenced by asymmetry	Age group 16–25
Hwang et al. [19]	2016	120	Relative eyebrow width/relative medial midpupilary and lateral heights of eyebrows to length of palpebral fissure measure over last century from photographs in Vogue magazine	Observational level IV	REW unchanged RLH greater than REW over time	Cross cultural difficult to compare
Galantucci et al. [20]	2016	66	25 anatomical landmarks total of 5610 data items	Cross-sectional level IV	Greatest influences on attractiveness are facial width, upper facial convexity; distance between nasion and midpoint of tragi; nasolabial angles and mouth width	Three-dimensional anthropometric analysis to set up a database statistically significant differences only in some measurements.
Heidekrueger et al. [13]	2016	1011	Lip shape preference	Survey level IV	Non-caucasian surgeon prefer larger lips and caucasian surgeons prefer smaller lips	14% response rate
Murakami et al. [21]	2016	9 morphed facial types	Lip position	Observational level IV	Favoured lip position differed between lay person and clinician	Japanese population—limited to specific ethnicity
Bagheri et al. [62]	2016	200	Lip morphology	Case control level III	Medium and full lip preference in males and medium and thin preference in females	Anatolian females computer-assisted redesign solution for lip augmentation
Tauk et al. [22]	2016	18	Visual Analogue Scale	Cross-sectional level IV	Entire face profile used to assess beauty	
Forte et al. [29]	2015	66	Attractiveness and tiredness on a 0–10 scale with digital alteration of facial subunits	Survey level IV	Neck ptosis, jowels, vertical lip rhytids, crows’ feet lower lid herniation influenced perception of age	Perception of tiredness and attractiveness extrapolated from impact on age
Alam et al. [60]	2015	286	Comparison to golden ration	Cross-sectional level IV	Only 17.1% conform to the ratio. 54% have shorter face. No association between golden ratio and facial evaluation scores	Malaysian population
Gibelli et al. [23]	2015	40	Lip measurements and differences in gender and age	Cross-sectional level IV	Male lips larger than female. Younger people have larger lips than older. Lower lip thickness highest percentage if correct for age	Three-dimensional technology used for morphological and metrical analysis
Penna et al. [24]	2015	176	Lip morphology	Cross-sectional level IV	High ratio of upper vermillion height to mouth–nose distance and chin–nose distance in and wider vermillion height/chin–mouth distance in attractive females	250 voluntary judges through an Internet presentation
Wu et al. [25]	2015	80 patients 50 landmarks	Facial characteristics	Case control level III	Attractive men had large forehead reduced mandible round baby face characteristics	Consider individual faces—Chinese population
Farrera et al. [26]	2015	565 patients	Asymmetry	Cross-sectional level IV	Attractiveness and asymmetry are not correlated	Use two-dimensional digital photographs and geometric morphometric methods Mexican population
Bronfman et al. [41]	2015	13 studies	Facial distances, angles and features	Systematic review of level III trials level III	Japanese adults have less bilabial protrusion, less prominent nose. Japanese adults prefer a more retruded profile	Used some skeletal measurements
Hwang et al. [19]	2015	37	Eye measurements	Cross-sectional level IV	Beautiful women and femme fatales have same inter-pupillary distance	Western society
Hwang et al. [27]	2014	31	43 distances and angles in young and old Leonardo’s profile drawings	Cross-sectional level IV	39 anthropometric items did not differ. Upper lip height, upper face height and nasolabial angle greater in young.	Comparing old and ‘ugly’ with young and beautiful
Park et al. [28]	2013	52	17 anthropometric ratios	Observational level IV	Femme fatales had narrow noses and attractive midface	Comparison of portrait paintings
Rosetti et al. [30]	2013	400	Facial distances	Observational level IV	Eye–mouth distance/height of mandible ratio influenced by attractiveness. Most facial ratios differ from golden ratio	Three-dimensional facial distances used
Wong et al. [31]	2010	197	Lip measurements and subjective assessment of attractiveness in different ethnicities	Observational level IV	Smaller than average in midline upper lip rated more attractive. Ethnic differences	Three-dimensional facial distances used. Lips did not contribute to attractiveness as much as previously thought
Pancherz et al. [32]	2010	158	5 transverse and 7 vertical measures compared with PHI	Observational level IV	Attractive individuals have proportions close to PHI	Testing Ricketts’ hypothesis
Pallett et al. [33]	2010	122 raters	Eye mouth distance intraocular distance	Survey level IV	Vertical distance between eyes and mouth = 36% of length horizontal distance between eyes is = 46% of width	Attempt to redefine ‘new’ golden ratio
Komori et al. [34]	2009	114	Averageness and symmetry	Observational level IV	Males and females both averageness and symmetry rate positive, whereas in female only averageness does	
Jahanbin et al. [35]	2008	50	5 landmarks 5 ratios	Cross-sectional level IV	Only some measures conform to the divine proportion	Use two-diemensional digital photographs
Holland [36]	2008	0	Analysis of the Marquardt’s mask	Observational level IV	Marquardt’s mask described as ‘not ideal’	
Medici et al. [37]	2007	20 digital images	Ratios of facial features rated by 12 individuals	Survey level IV	A relationship exists between divine proportion and aesthetic face	Manipulation of ratios by morphing from 2.0 to the divine ratio
Danel et al. [49]	2007	77	Eye mouth angle	Observational level IV	Attractiveness negative correlation to EME	
Kim et al. [38]	2007	40	Rating of pre and post-operative photographs with the Marquardt mask	Observational level IV	Results not statistically significant but mask a ‘useful’ tool	
Costa et al. [39]	2006	1065	Eye lip size and roundness	Case Control level III	Eye and lip roundness, eye height and width and lip height are enhanced in artistic portraits compared to photographic	One part of three studies
Milutinovic et al. [40]	2014	107	Facial distances and proportions	Observational level IV	Smaller face/uniformity of thirds and fifths and most parameters meet the ‘ideal proportions’ in aesthetically pleasing faces	
Gan et al. [54]	2014	307	Self-taught learning computer based	Cohort level III	Facial beauty can be recognised at a rate 87.3% of face	
Xie et al. [55]	2015	500	Benchmarking the SCUT-FBP dataset	Case control level III	Confirming the SCUT-FBP dataset is reliable for predicting attractiveness	

## Discussion

Measurements of facial proportions introduced by the Greeks, the Classical Canons and later adopted by the Renaissance artists, the Neoclassical Canons are used by surgeons today to understand ideals of beauty and reproduce aesthetically ‘beautiful’ proportions for patients. To date, there is no agreed standard to measure facial beauty, and this remains a challenging task. It is a vital consideration for the aesthetic surgeon because there is a positive association between the outcomes of aesthetic surgery and better mental and psychological health, and therefore measuring outcomes quantitatively would facilitate this endeavour [42]. The aim of this investigation was to undertake a review to answer the question.

In the treatment of adults requesting facial aesthetic improvement, is there an evidence-based approach in quantitatively assessing beauty that is useful in everyday aesthetic practice?

Upon reviewing the data, it became evident that the diverse measurement criteria, methodologies used and population types in trials made it difficult to compare data. For example, different measurements from different types of photographic techniques would introduce photographic bias [43]. The trials have at best been of Level III or less for evidence, mainly being cross-sectional studies or observational studies. Despite these difficulties, some common themes were discovered and are highlighted below. These were related to lip analyses, eye measurements, symmetry, ethnicity, automation of analysis and the golden ratio.

### Lip Measurements

Lip augmentation is one of the most common aesthetic procedures undertaken to correct age-related changes [44]. In the perception of beauty and attractiveness, measurements for individual facial features have been used. Lip measurements, for example, are known to influence an aesthetically youthful appearance [45]. Some authors define an ‘ideal lip’ as having good definition of the vermillion border with lower and upper lip balance [46].

Bagheri et al. undertook lip measurements in a Turkish population of 200 persons who were classified into of full, medium, thin and very thin type lips. They concluded there were significant gender differences in lip sizes and the aesthetic ranking of lips. Medium and full lip types were the significant proportion in males, and in females, medium was predominant. They also concluded that very thin lip types are rare in both sexes [62]. Heidekrueger et al. undertook a cross-sectional analysis of lip size preference through an online survey of 9000 plastic surgeons and lay persons. With a response rate of 14% from 35 different countries, they suggested the ethnicity, country of residence and profession had an impact on lip shape preference. They found that surgeons, who are non-Caucasian or who practise in Asia, have a preference for larger lips, whereas European and Caucasians prefer smaller lips. In the follow-up of this paper using the same responses, the team was able to assess the most popular lip ratio in the survey takers. A ratio of 1:1 was preferred in 60% of responders, whereas Popenko et al., assessing the attractiveness ranking of lip dimensions in 100 morphed faces of Caucasian women, suggested 53.5% increase in surface area from baseline and 2:1 ratio of lower to upper lip was the more attractive. Penna et al. found there was a higher ratio of upper vermillion height to mouth–nose distance and of chin–nose distance in attractive females.

These studies are not directly comparable due to the diverse measurements and populations but give us an insight into some lip preferences of patients and surgeons.

### Eyes

Eye size, position, eyelid ptosis and eye ‘frame’ have all been related to perceptions of beauty.

Bensilmane introduced ‘the Frame concept’ to quantify and assess the characteristics of the female periorbital region. The author highlighted the fact that aesthetic practitioners most often analyse both upper and lower lids separately and rarely the gaze itself. The author strives to validate this ratio using anthropometric measures, to prove his hypothesis that the narrower the frame the more aesthetically pleasing [16]. As Benslimane et al. validate ‘the frame’ concept, the authors agree that a jaguar-like upward slant of the lower eyelid is more pleasing [47]. Photographs of models were analysed and frame anthropometry measured. The frame height was found to be inversely correlated with attractiveness, and this was synonymous for classical portraits of beautiful females.

Costa et al. reviewed photographs and historic artistic portraits and established that eye roundness, height and length were enhanced in artistic portraits, suggesting these features may be more beautiful. Larger eye size in proportion to the face has been shown to be more attractive in females [48]. Danel et al. identified that the eye–mouth–eye (EME) angle can be used as a quantitative measure of masculinity and fascial symmetry in males, which is independent of facial size. They concluded that there is a negative relationship between the EME angle and attractiveness. Hence, eye size, eyelid ptosis and frame contribute to beauty with a negative correlation with EME angle [49].

### Symmetry

Kaipainen et al. assessed regional facial asymmetry and its influence on attractiveness. In their small sample size, most had some facial asymmetry, particularly in the lower and middle third of the face. The team did not find any association between regional asymmetry and attractiveness. Komori et al. suggested that the female facial symmetry does not appear to affect attractiveness. Their sample size was small, and therefore their conclusions may not be extrapolated. Farrera et al. undertook measurements from photographs of 565 Mexican individuals and chose a sample of 100, to rate for attractiveness after grouping into asymmetry variation. Their conclusion was also that symmetry does not affect attractiveness.

Other observers suggested that symmetry is important to facial beauty [50, 51]. Honn et al., for instance, argued that the symmetry has an influence on attractiveness [52].

Scientists and philosophers have traditionally attempted to appreciate attractiveness and beauty in terms of symmetry, and therefore, it may be that symmetry is not as important as previously thought, and perhaps beauty is related to proportions or ratios of the facial aesthetic units rather than to symmetry [53].

### Ethnicity

We live in a heterogeneous society with persons from diverse backgrounds seeking aesthetic treatments. It is therefore important that the clinician is aware of average facial characteristics of different ethnic groups.

Bronfman’s systematic review looked at 13 different studies focusing on the Japanese preference for aesthetic profile and concluded that Japanese males had smaller noses and bilabial protrusion, whereas females had more bilabial protrusion and a less prominent chin when compared with white populations. American and Japanese examiners favoured a lip profile that was retruded compared to African examiners. This suggests that consideration should be given to the ethnicity of the patient and that the clinician should be aware that one’s own ethnic background may have an influence on the shared decision making during a consultation for aesthetic treatment.

### Technology and/or Automation

Geometric evaluation of features and proportions is cumbersome and requires considerable investment of time. If the measurements can be predicted or calculated by software, a more rapid appraisal of beauty in the clinic setting is possible.

Gan et al. introduced a novel method for extracting facial features from images using an algorithm through machine learning [54]. This approach may avoid the likelihood of manual intervention. Although the idea of automated beauty recognition is novel, his paper utilizes simple non-detailed information such as curves and edges and is not concerned with the individual facial structures such as the eyebrows and nose. This prediction, however, is based on two-dimensional photographs, possibly lending itself to measurement inaccuracies and photograph bias. Xie et al. argued the case for a dataset of geometric measurements for application in facial beauty analysis [55] They gave attractiveness ratings using classical and deep learning methods to develop an algorithm, to learn and to predict facial beauty automatically. Galantucci et al., on the other hand, set out to verify a facial beauty prediction modelling method of principal component analysis (PCA) for measuring facial features for beauty classification [56]. The team used three-dimensional digital photogrammetry on real Miss Italy 2010 beauty contestants to confirm beauty ranking and PCA analysis to conclude that it is not a valid prediction tool. Mojallal et al.’s commentary paper appreciates the value of quantitative measurement of volume loss through the use of a three-dimensional camera. The team argued that the classical anthropometry measurements of the face are highly inaccurate and the ‘differences in volume, distance, and projections’ are too small to measure by these methods. They suggest objective evaluation, through the use of digital three-dimensional stereophotogrammetry, after facial rejuvenation allowing 360° views of the individual. The advantage of this technique is that it allows for volume measurement as well as proportion measurement. The disadvantages are that the images require special manipulation and the technique is time consuming, and therefore it may not actually be useful in everyday practice [57]. Rossetti et al. also employed the use of three-dimensional stereophotogrammetry to investigate whether the ‘golden’ relationship exists between measurements of facial features [58]. They undertook measurements using reproducible three-dimensional techniques already described and validated [59]. Multiple measurements were undertaken, and the authors used previously acknowledged ‘traditional’ landmarks. Through their statistical analysis of the measurements, the team used ten ratios to compare with the golden ratio. Their analysis concluded there was no similarity to the golden ratio in their measurements.

Their study is important because they utilise three-dimensional technology for measurements, allowing easy calculation of the distance between facial landmarks. This gives a highly representative sample to work with. Three-dimensional stereophotogrammetry in their study was useful to undermine the theory of the golden proportion, or golden ratio, in most measurements of facial proportion though it cannot be extrapolated to other ethnic groups.

### The Golden Ratio

The concept of the golden ratio has been used since the time of Phidias, and its relation to aesthetic beauty still continues to be debated.

Alam et al. investigated the association of facial measurements with the golden ratio in a Malaysian population [60]. Using direct facial measurements from surface landmarks rather than from photographs is possibly more accurate. This cross-sectional study of 286 patients found that, in this population, only 17.1% of facial proportions correlated with the golden ratio and concluded that an association does not exist between the facial measurements in their Malaysian population and the golden ratio. Jahanbin et al. used 50 standardised profile silhouette photographs and 20 judges scored these on a VAS score. Measurements were made to assess whether any facial proportions fit the golden ratio. After assessing five landmarks and five ratios, none had the golden ratio mean of 1.618. Park et al., testing 17 anthropometric ratios for portraits of femme fatales, showed a midface ratio of 36% of the total face height. The proportions from portraits are closer to ‘ideal’ than in ratios measured clinically. Jang et al. undertook measurements from three-dimensional sampling of 93 patients in a Korean population and concluded that a longer face, smaller lip and chin size were preferable in females of a Korean population and this is a deviation from the golden ratio [61]. Milutinovic et al. assess different facial proportions and their relationship to attractiveness in Caucasians and any deviations from the ideal proportions or the divine ratio. In their group, they found that in attractive females, the divine ratio was met in three out of the six measured parameters. They establish that attractive females have facial proportions nearer to the divine ratio.

Medici et al. examined four ratios of frontal photographs of 20 Caucasian patients and concluded there is a relationship between the divine proportion close to or at a ratio of 1:1.618 and facial aesthetics. Kim et al. assessed the usefulness of the golden ratio and application through the use of the Marquardt’s mask in forty cases of pre- and post-operative photographs. Scores were compared for the applied mask and for those without applied photographs and concluded that the Marquardt’s mask was useful as an analytical tool for facial analysis, whereas Holland argued in his article that the Marquardt’s mask is less than ideal. He states the methodology used to assess the fit of the mask for faces is ‘faulty’, that the mask approximates to a masculinised European female face and that it does not appear to approximate to a desired ‘ideal’ face. Undoubtedly, all this evidence suggests that the jury is still out on the usefulness of the golden ratio and that a consensus does not yet exist on this issue.

## Conclusions

There is an overwhelming desire to quantify beauty when planning aesthetic procedures in the light of increasing demand, and therefore an evidence-based approach is desirable. From the Greek scholars, through to the Renaissance polymaths, to today’s three-dimensional predictive modelling, we have attempted to define and measure beauty. Despite measures of individual components such as fuller lip size and defined vermillion border, larger eyes and the ‘Frame concept of eyes’, through to the sum of all the parts, symmetry and the Golden Ratio, we are yet far from establishing what truly constitutes quantitative beauty.

It may ultimately be the case that measuring beauty may not provide great practical value because comparative measurements ignore the individuality in all of us. Perhaps as the famous poet Margaret Hungerford states that beauty is truly in the ‘eyes of the beholder’.
